# ‘It's a girl!’ Is gender disappointment a mental health or sociocultural issue?

**DOI:** 10.1192/bjb.2023.78

**Published:** 2024-12

**Authors:** Chrissy Jayarajah

**Affiliations:** Central and North-West London Foundation NHS Trust, London, UK

**Keywords:** Adjustment disorders, perinatal psychiatry, transcultural psychiatry, stigma and discrimination, gender incongruence and gender dysphoria

## Abstract

Gender disappointment can be defined as subjective feelings of sadness when discovering that the sex/gender of a child is the opposite of what the parent had hoped or expected. Wanting a boy (or ‘son preference’) has long been noted in many cultures, particularly in South and East Asian communities, but it is now becoming more recognised in the UK, Europe and North America. This article aims to improve understanding of gender disappointment by exploring medical and social sciences research; it also discusses the clinical and risk implications of assessing and managing gender disappointment (or not doing so) when individuals present to perinatal and/or community mental health services.

## Disclaimer

The terms ‘sex’ and ‘gender’ are used interchangeably in this article in keeping with inconsistencies noted in the limited evidence-based literature on this topic over the past 20 years. Both terms are used here in reference to traditional beliefs about the biological determinants of sex and to the social definition of gender assigned at birth. I acknowledge the recent wider cultural and political discussions surrounding gender, namely the more modern understanding and beliefs about gender fluidity and non-binary identification.^1^ Similarly, the term ‘mother’ is used predominantly in this text to echo its use in the literature, but I acknowledge that gender disappointment may occur in any birthing person and/or primary caregiver – although unfortunately much of the evidence base is not reflective of current commentary and conversations happening in a wider sphere.

## What is gender disappointment?

Gender disappointment can be defined as subjective feelings of sadness when discovering that the sex/gender of a child is the opposite of what the parent had hoped or expected. Wanting a boy (or ‘son preference’ as referred to in the literature) has been noted for generations, particularly in South and East Asian communities, but it is not classified as a diagnosable mental illness. In recent years, there has been increased interest in this subject, specifically the medicalisation of the associated emotional discomfort and distress, which may have significant sociopolitical ramifications. This article aims to improve understanding of gender disappointment and address the question of whether it is helpful to see it as a mental health or a sociocultural issue. Finally, I discuss how best to assess and manage such issues when they arise in perinatal and/or community mental health services.

## ‘Unnatural’ selection

Although there are numerous articles, blogs and forums discussing the notion of ‘gender disappointment’, there is comparatively little in the way of formal evidence-based literature on the subject. Online forums focus on the dilemma of a mother who already has one, two or more children of the same sex (all girls, for example) and feelings of disappointment on finding out she is expecting another girl (or *vice versa* in a family of boys). The strong anticipative desire (and subjective let-down) is often commented on by others, asking whether they will ‘try again’ for a boy/girl. Gender disappointment in Western cultures is mostly related to the desire for ‘gender balance’ in the family – having a child of each gender to experience being a mother to sons as well as daughters.

Preferences for gender are more common than one may think. Parents are often subject to (unsolicited) advice in the form of old wives’ tales for guaranteed success in ‘choosing’ their baby's gender. ‘Natural methods’ promising to influence the sex of the baby include specific dietary modifications, the Chinese gender predictor chart and strict instructions on how to conceive (such as various sexual positions and timings of intercourse).^2^ Although these methods may appear extreme to some, gender selection is fast becoming a growing business. The ability to find out the gender of the baby before birth is a relatively new phenomenon, due to the rapid development of sonography in the 1980s, which allowed gender to be determined during the anomaly scan at approximately 20 weeks’ gestation.^3^ Thus, the current generation of expecting parents have the option of finding out half way through their pregnancy, and those who are keen to know sooner can pay for the privilege of non-invasive prenatal testing (NIPT), which can determine gender through a simple blood test as early as 9 weeks into the pregnancy.

For those who want to know the sex even before conception, this is possible via pre-implantation genetic diagnosis screening through the process of *in vitro* fertilisation (IVF). This screening (which is primarily used to identify gender-related disorders and check embryo health, but has a secondary gain of learning the sex of the embryo) is permitted only in limited circumstances in the UK, where embryo selection is legal only to avoid serious inherited illnesses, but for those willing and able there are a number of clinics around the world that provide this service at a cost.^4^ The cost, however, may be more than just financial. Prenatal sex selection against females (PNSSaF) has led to more than 100 million ‘missing’ girls across Asia, with more recent studies showing that it is more geographically widespread among the Asian diaspora across the UK, USA, Canada and Europe.^5^ There are several ethical and political considerations that factor into this debate about the future of sex selection and whether this will be the gateway to selection of other phenotypes.

## Gender disappointment – a parental problem?

The concept of gender and gender identity remains a hot and controversial issue. There has been an increased awareness and conversations about gender, which may contribute to increased reflections and hyperfocus on gender norms. Although this article is selective to the discussion of gender disappointment (with the specific definition given above, related to discovering the sex of a child during pregnancy or at the time of birth), I acknowledge there is a wider discourse on gender which may occur later in a child's life, particularly if parents are disapproving of the child's sexual orientation or indeed ‘disappointed’ if the child or young adult decides to change their gender/sex assigned at birth. Gender disappointment can also be experienced by individuals themselves (for example in those in the trans or non-binary community), many of whom describe feeling ‘trapped’ inside the ‘wrong’ body and gender. Both these scenarios, although not strictly within the scope of gender disappointment as defined in the literature, are important to mention as they can have significant implications for individuals and families as whole, who may experience discrimination, conflict and rejection and also mental health difficulties if not conforming to the mainstream binary views on sex and gender.^6^

## Western influences – gender reveals and revelations

One possible explanation for gender disappointment in Western culture is that of the emerging trend for the idealisation of gender, particularly in the wake of social media. We have seen the rise in popularity of ‘pregnancy announcements’ made on online platforms as well as ‘gender reveal’ parties, with their videos becoming viral. The societal shift may relate to the emergence of the idealisation of motherhood in a culture preoccupied with parenting perfectionism. Mothering through the (albeit virtual) gaze of others can be a double-edged sword. For many, it provides a sense of community and affirmation of a new identity (during what can be a happy but also challenging transition, commonly referred to as matrescence). It can, however, spark insecurities, particularly for those with preexisting mental disorders and/or those who struggle with comparison and may feel unsure that they are, as Donald Winnicott famously described it, a ‘good enough mother’, let alone perfect.^7^

In addition to the social pressures online, there may also be pressures within the person, as the whole process of bringing life into the world will naturally cause the individual to reflect on their own upbringing, as well as their ideas, beliefs and values regarding gender and family. We are seeing an increase in this trend in countries such as the USA, UK, Canada and Australia, where second- and third-generation women of various ethnic minorities are faced with the expectations of their traditional cultural upbringing, combined with the Western influence of a parenting culture with fixation on gender-normative values.

## Eastern influences – son preference (or is it daughter discrimination?)

If we take a closer look at the literature from the Asian and Indian subcontinent, we see that the term ‘gender disappointment’ is virtually non-existent. A literature search for ‘son preference’, however, generates thousands of references, which presumably reflects cultural variations in attitudes to having a son, especially as a first-born child. Possible reasons for son preference include belief that it will create financial stability through continuing the family inheritance of wealth and receipt of dowry (financial payment to the family on marriage of the son to a bride). There is also a weighted emphasis on traditional gender roles, identity and expectations (men are seen as powerful, independent and dominant, whereas women are seen as nurturers, putting family obligations first).^8^ Sociologists assert that these beliefs in the high value of men as the cornerstone of a patriarchal society are ingrained, passed down through families via modelling (social learning) as well as the internalisation of social ideas and values as personal ideas and values (self-determination) – namely, women accepting their subordinate place in society and, by doing so, aiding preservation of the culture.^9,10^

In East, Central and South Asian communities societal structure is firmly imprinted on patriarchal and agricultural values. Having a boy equates to economic and social stability (for continuing both the family name and business, which may be labour intensive or requiring additional qualifications – both of which are deemed to be ‘male appropriate’). Having a girl is considered a social and economic disadvantage in many low- and middle-income countries, as the luxuries of education and employment may not be as readily available to females, thus limiting their earning capacity and/or potential to generate wealth. Rawat et al also noted that for mothers in rural India, gender preference was significantly associated with religion (Hindu or Islamic faith), the earning status of the mother, socioeconomic status, the gender of the previous child and age of the mother at marriage.^11^

Theerthaana et al acknowledge the importance of social and familial influences, but also recognise the pivotal role of within-couple dynamics and individual personality structures. They found that gender disappointment in new and expectant mothers may be strongly influenced by their husbands’ desire for a son and lack of emotional and/or practical support in having a girl – drawing on theories of gender discrimination and parental investment theory (the cost analysis comparison of having sons/daughters) as discussed above. Interestingly, in their survey of over 3000 mothers in India, different personality structures were shown to influence gender disappointment through different mechanisms. Neuroticism was associated with increased postnatal anxiety/depression and it moderated the response to the familial, cultural and social expectations. Those with extraverted personality types may be prone to gender disappointment should the gender reveal have a negative impact on their perceived societal and economic status, and those with a conscientious personality structure (who by nature are highly methodological and organised) may worry about the financial implications of raising a daughter and the additional safety concerns associated with gender discrimination.^12^

Interestingly, the literature suggests that in Western culture there is a notable daughter preference, due to strong inherited beliefs that girls are innately more empathetic, emotionally aware, nurturing and closer to the family, in comparison with boys, who are seen as independent, less emotional and for whom it is more acceptable to ‘fly the nest’. These same qualities are listed for son preference in Eastern and Asian cultures, highlighting the same gender stereotypes but different preferences depending on where you live and your family of origin.^13^

## East, West or global concern?

On broadening the literature search to include both medical and social science literature, we begin to see the complexity surrounding gender disappointment. It is far too simplistic to suggest that gender disappointment is an ‘East versus West’ or even ‘boy versus girl’ divide. Thanks to immigration and the internet, interracial and multicultural families and communities are now the norm in many towns and cities across the world, London being an example of this, where more than 250 languages are spoken.^14^ In clinical practice, we have seen a rise in concerns about gender disappointment from women of a variety of backgrounds, including Pakistani, Chinese, Afghani and American. Women with daughters, particularly from Asian and Arab communities, have talked openly about the ‘pity’ received from other women, as well as the shame felt for not having a boy. These beliefs are more common across the Middle East, as discussed in a study exploring fertility and family planning in the Arab region, which noted that the average number of children is higher compared with other parts of the world (from four in Egypt to nine in Mauritius).^15^ Greater contraception use was recorded not only among those with higher socioeconomic backgrounds living in urban areas, but also among women who had more than three children and more sons than daughters, implying that gender at birth can influence the use of birth control and thus the overall birth rate and fertility levels.

## Could gender disappointment be evidence of psychopathology?

Although the argument regarding gender disappointment and/or son preference from a sociocultural perspective may be convincing, the notion of identifying it as a treatable mental illness is less so. There has been a discourse brewing within and around psychiatry for many years about the fine line between normality and pathology, with the potential for serious harm by ‘medicalisation’ of what some have argued to be the normal continuum of human experience and emotion. Although it is beyond the scope of this article to explore this argument fully, psychiatrists such as Pat Bracken and Joanna Moncrieff have been instrumental in creating a space for discussion in the critical psychiatry sphere – specifically, core beliefs in psychiatry and the (over?) use of psychotropic medications – while embracing novel approaches to understanding mental health needs through the open dialogue approach.^16^ Hendl et al argue that gender disappointment should not be considered a distinct category of mental illness and they warn of the pitfalls of allowing societal attitudes to have a too powerful influence on defining mental illness – with the stark reminder that homosexuality was classified as a mental disorder in the DSM up until 1973. They propose that the main issue lies in ‘gender essentialism’ – the belief that only children of a particular sex are capable of certain gendered activities, personality traits and stereotyped behaviours (girls liking ballet and boys playing football, for example).^17^

Despite this, there is growing demand for gender disappointment to be recognised as a diagnosable (and thus treatable) condition, which could have serious implications. First, in the guise of being ‘socioculturally sensitive’, it may risk strengthening claims for medical help and support for choices that may be both sexist and illegal under UK abortion laws. Second, the medicalisation of this phenomenon could lead to missing and/or misdiagnosing of other mental health conditions presenting in the perinatal period. Examples include postnatal depression (due to feelings of maternal ‘incompleteness’, guilt and sadness), perinatal obsessive–compulsive disorder (due to excessive ruminations about sex preference), post-traumatic stress disorder (relating to the perceived trauma of not having the desired parenting experience, usually after multiple children of the same sex) or likening it to a grief reaction, mourning for ‘something you never had’. Indeed, none of these symptoms in isolation would classify as a mental disorder, but any one of these psychiatric conditions can overlap and/or coexist alongside feelings of gender disappointment.

## Assessment of gender disappointment

As in assessment and treatment of any significant trauma or normal grief reaction, there is the potential for gender disappointment to reach the threshold for a mental disorder, but one would be advised to wait for at least 3 months before making any assumptions let alone giving a diagnostic label and/or considering psychiatric or psychological treatment. Waiting 3 months, however, may be problematic during the perinatal period, because if low mood and/or gender disappointment are not both recognised and managed in a timely way they can spiral and affect maternal bonding and infant attachment. As regards assessment, as with any patient seen during the perinatal period (currently defined as from the moment of conception up to 1 year postpartum), one must pay particular attention to the details of the pregnancy (for example, was this a planned/unplanned pregnancy or welcome/unwelcome ‘surprise’?). It is crucial to assess maternal bonding, clues of which start even while the baby is *in utero* and may predict the direction following delivery. Although there are a few reliable tools available to assess maternal attachment and bonding, simple screening questions while in clinic such as asking how the individual feels about the pregnancy/baby, whether they sing or talk to the unborn child and whether they have started to prepare for the baby's arrival are a good starting point. One should also be mindful of the beliefs and attitudes of the mother to be, their partner and the family culture of origin (particularly if they are of an ethnic minority) and whether the patient is multiparous and has two or more children of one sex and/or history of repeated terminations.

## Management of gender disappointment

It is important to note that gender disappointment can present to any healthcare professional in community or acute settings. Clinicians have noted that most uncomplicated gender disappointment presents as mild and self-limiting, resolving soon after birth without the need for intervention (as the act of seeing and holding the healthy baby in their arms is often enough for most to banish previous disappoint or doubt); however, some individuals may require more intensive support, often psychological in nature, in preparation for managing expectations on arrival of the newborn, with careful monitoring thereafter of the mother–child relationship.^18^ For those with more complex and/or comorbid psychiatric presentations, these should be identified and treated accordingly, ideally in specialist perinatal mental health services. By taking a holistic approach, this may be a good opportunity to gain a greater understanding of beliefs about gender and to increase awareness of the possible medico-legal risks of requests for late termination relating to gender disapointment (further information available from the author on reasonable request).

## Conclusions

Historically, gender disappointment has rarely made it to the attention of secondary care mental health services owing to the subclinical nature of its presentation. Things are, however, slowly changing. The shift towards community-based mental health services in the past two decades, coupled with the more recent expansion of perinatal mental health services across the UK, has led to greater opportunities for awareness, assessment and treatment of psychological and psychiatric problems during pregnancy and the postnatal period.^19^ At present, there is little dialogue about gender disappointment, which has led to misunderstanding and the potential for serious repercussions. My hope is that this article may act as a catalyst for a nuanced discussion at a local and national level, as well as ignite interest in more evidence-based research on gender issues in mental health.

## Data Availability

Data availability is not applicable to this article as no new data were created or analysed in this study.

## About the author

**Chrissy Jayarajah**, MBBS, MRCPSYCH, DFSRH, is a consultant perinatal psychiatrist and clinical lead for Central and North West London Foundation NHS Trust Perinatal Mental Health Services, and an honorary senior clinical lecturer at Imperial College London, UK. She also holds a Diploma in Sexual and Reproductive Health from the Royal College of Obstetrics and Gynaecology's Faculty of Sexual and Reproductive Health.
